# ‘*Candidatus* Liberibacter asiaticus’, Causal Agent of Citrus Huanglongbing, Is Reduced by Treatment with Brassinosteroids

**DOI:** 10.1371/journal.pone.0146223

**Published:** 2016-01-05

**Authors:** Eduardo Canales, Yamilet Coll, Ingrid Hernández, Roxana Portieles, Mayra Rodríguez García, Yunior López, Miguel Aranguren, Eugenio Alonso, Roger Delgado, Maritza Luis, Lochy Batista, Camilo Paredes, Meilyn Rodríguez, Merardo Pujol, María Elena Ochagavia, Viviana Falcón, Ryohei Terauchi, Hideo Matsumura, Camilo Ayra-Pardo, Raixa Llauger, María del Carmen Pérez, Mirian Núñez, Melissa S. Borrusch, Jonathan D. Walton, Yussuan Silva, Eulogio Pimentel, Carlos Borroto, Orlando Borrás-Hidalgo

**Affiliations:** 1 Department of Plant Science, Center for Genetic Engineering and Biotechnology, Havana, Cuba; 2 Chemistry Faculty, University of Havana, Havana, Cuba; 3 Group of Phytopathology, Instituto de Investigaciones en Fruticultura Tropical, Havana, Cuba; 4 Empresa Agroindustrial Victoria de Girón, Jagüey Grande, Matanzas, Cuba; 5 Department of Genomics and Breeding, Iwate Biotechnology Research Center, Kitakami, Japan; 6 Gene Research Center, Shinshu University, Ueda, Japan; 7 Department of Physiology, National Institute of Agricultural Science, Mayabeque, Cuba; 8 Department of Energy Plant Research Laboratory, Michigan State University, East Lansing, Michigan, 48824, United States of America; 9 Group of Phytopathology, Tobacco Research Institute, Havana, Cuba; UMBC, UNITED STATES

## Abstract

Huanglongbing (HLB) constitutes the most destructive disease of citrus worldwide, yet no established efficient management measures exist for it. Brassinosteroids, a family of plant steroidal compounds, are essential for plant growth, development and stress tolerance. As a possible control strategy for HLB, epibrassinolide was applied to as a foliar spray to citrus plants infected with the causal agent of HLB, ‘*Candidatus* Liberibacter asiaticus’. The bacterial titers were reduced after treatment with epibrassinolide under both greenhouse and field conditions but were stronger in the greenhouse. Known defense genes were induced in leaves by epibrassinolide. With the SuperSAGE technology combined with next generation sequencing, induction of genes known to be associated with defense response to bacteria and hormone transduction pathways were identified. The results demonstrate that epibrassinolide may provide a useful tool for the management of HLB.

## Introduction

Citrus Huanglongbing (HLB), previously known as citrus greening, constitutes the most economically devastating disease of citrus worldwide, being present in around 40 countries [1]. HLB causes severe symptoms, depending on the age and the number of infections per plant [2, 3]. The disease is caused by the fastidious gram-negative α-proteobacteria *Candidatus* Liberibacter asiaticus (Las), *Ca*. L. africanus, and *Ca*. L. americanus as well as some phytoplasmas. The bacterium propagates within the phloem of citrus plants producing die-back, yellow shoots, blotchy mottles on leaves, and off-tasting and malformed fruit [4, 5]. The ‘*Ca*. Liberibacter’ bacterium is transmitted by the psyllids *Trioza erytreae* in Africa [6] and *Diaphorina citri* in Asia and the Americas [7, 8].

Management of HLB in commercial areas is limited to controlling the psyllid vectors with insecticides, removal of symptomatic trees to reduce inoculum levels, geographical isolation, and certification of propagation of pathogen-free budwood sources and nursery trees [5, 9]. The development of resistant citrus cultivars would constitute the most efficient control for HLB, but, unfortunately, resistance genes have not been identified for use in breeding programs [5, 10]. Ultimately, development of methods for genetic transformation of the citrus plant, the pathogen, and/or the vector could lead to disease-resistant cultivars and /or reduction of the bacterial pathogen and insect vector [3, 11]. Since many citrus trees are already infected, it is essential to develop an efficient treatment to combat HLB. One attempted approach to control HLB has been the use of compounds that induce systemic acquired resistance (SAR), such as salicylic acid. However, no significant difference was seen between treated and untreated plants, probably because the bacterium has an enzyme that degrades salicylic acid [3, 9].

Brassinosteroids are plant hormones that regulate multiple developmental and physiological processes, including seed germination, stem elongation, leaf expansion, xylem differentiation, disease resistance, and stress tolerance [12, 13]. Brassinosteroids are sensed by a leucine-rich repeat receptor-like kinase (LRR-RLK) (BRI1) that is localized in the plasma membrane [14]. Binding of brassinosteroids to pre-existing BRI1 leads to BRI1 autophosphorylation and to transphosphorylation of the brassinosteroid-associated kinase 1 (BAK1), a member of the SERK (Somatic Embryogenesis Receptor Kinase) family [15].

The connection of brassinosteroids to plant defense was suggested by the association of BAK1 with both pathogen-associated molecular patterns (PAMP)-triggered immunity (PTI) responses and brassinosteroid signaling [16–18]. BAK1 is involved in signaling mediated by FLS2 and EFR [19], two LRR-RLKs associated with recognition of pathogen molecules. In both cases, BAK1 acts as a co-receptor and is required for signal transduction. Although brassinosteroid treatment increases resistance to a wide range of pathogens [20–28], the role of brassinosteroids in plant responses to pathogens is complicated and the regulation of immunity occurs at multiple levels [18]. Currently, there is no evidence concerning the effect of brassinosteroids on citrus trees affected by HLB, nor how plant defenses in response to this important pathogen are modulated at the molecular level.

Development of therapeutic compounds for the control of HLB could provide an important solution for effective disease management. Here, we present evidence that 24-epibrassinolide (eBL) reduces ‘*Ca*. L. asiaticus’ bacterium titers in HLB-affected citrus plants.

## Materials and Methods

### eBL treatment of HLB-affected citrus under greenhouse conditions

Mexican lime (*Citrus aurantifolia* (Christm) Swingle) plants (2 years old and 1 meter in height) with typical HLB-associated symptoms were selected in a citrus production area affected by HLB. The plants were tested for titers of ‘*Ca*. L. asiaticus’ by using qPCR with specific primers and subsequently were dug out of the ground, placed into pots and moved to a greenhouse at 25°C with watering as needed for fruit production. The experiment took place from May, 2010 until May, 2011. Ten HLB-affected plants per treatment with similar initial bacterial titers were treated with two different concentrations (0.084 μM and 1 μM) of eBL (Ruina International, Zhengzhou) or water (control) every 15 days for 12 months. The experiment was designed with ten replicates per treatment. The bacterial titers were evaluated every three months during the experiment.

The canopies of the trees were sprayed to drip with ~100 ml of solution using a ZEP 32-ounce professional sprayer (Model HDPRO36, The Home Depot, Mexico). For each evaluation, a total of 10 leaf samples were collected from five different positions around the tree canopy. Three technical replicates were used for the qPCR analysis. Leaves with typical symptoms (blotchy mottle) were also sampled. The samples were picked and immediately placed in liquid nitrogen. After 12 months, all the leaves on the trees were tested to detect incomplete systemic distribution within the trees. Data were analyzed by an analysis of variance using GraphPad Prism Software Inc. (La Jolla, CA, USA). Significant differences among means were determined by two-way ANOVA with least significant difference mean separation at P < 0.05.

### eBL treatment of HLB-affected citrus under field conditions

Field studies were carried out at a citrus orchard in Matanzas, Cuba (geographical coordinates 22° 38.167’ N, 81° 16.749’ W). The experiment received the appropriate official permission from the enterprise authorities to conduct the study on this site. ‘Valencia’ sweet orange (*Citrus sinensis* (L.) Osb.) trees (2-years-old and 1.70 meters in height) were treated with 0.084 or 1 μM eBL or water (control) every 15 days for 12 months. The experiment was developed using a simple design (S1 Fig). Four rows of plants for each treatment with a total of 148 plants were used. The three first and last files of plants were left untreated to avoid any border effect. The eBL was applied with a mistblower TEYME 2090 http://www.teyme.es/en/equipos-agricolas/trailed-mistblower-unic-mh/ (Girona, Spain), with a capacity of 2000 liters. The final concentration of eBL was 0.04 mg/l (0.084 μM) and 0.48 mg/l (1 μM), respectively. To evaluate the reduction in titers, 15 HLB-affected trees per treatment were selected at random (S1 Fig). In each evaluation, a total of 10 leaf samples were collected from five positions around the canopy including leaves with typical symptoms (i.e., blotchy mottle). After picking, the samples were immediately placed in liquid nitrogen. The evaluation of ‘*Ca*. L. asiaticus’ bacteria titers using qPCR with specific primers was conducted at intervals of 3 months over 12 months. Additionally, 15 plants without symptoms and with no detectable bacterial titers per treatment were evaluated at the same time intervals. The fold-change was calculated by dividing the average of total bacterial titers before the eBL applications by the total titers after 12 months of applications. All the plants with and without typical symptoms of HLB prior to treatment were included in this analysis. The experiment took place from March, 2012 until March, 2013. Data were analyzed by analysis of variance using GraphPad Prism Software. Significant differences among means were determined by one-way ANOVA least significant difference mean separation at P < 0.05.

### DNA extraction and quantification of ‘Ca. L. asiaticus’

Leaves were washed under running tap water, blotted dry on filter paper, and their midribs cut into small pieces. Midribs (500 mg) were ground in a TissueLyser II homogenizer (Qiagen, Germany). Total DNA was extracted according to the method of Zhou *et al*. [29]. DNA preparations were adjusted to 100 ng DNA/μl and stored at -20°C. For real time PCR, the QuantiTect SYBR Green PCR Kit (Qiagen, Maryland, USA) was used. The SYBR Green reaction was performed in a 25 μl reaction mixture according to the manufacturer’s instructions. A Rotor—Gene 3000 PCR machine (Corbett, Australia) was used with the following program for DNA amplification: an initial 95°C denaturation step for 15 min followed by denaturation for 15 sec at 95°C, annealing for 30 sec at 58°C, and extension for 30 sec at 72°C for 40 cycles. For detection of ‘*Ca*. L. asiaticus’, the primer sequences (Las16S-forward: ctaatccccaaaagccatctc; Las16S-reverse: ggagttggttttgcctgaag) were designed according to Primer 3 online software (http://simgene.com/Primer3) using the 16S ribosomal RNA gene (LOCUS: FJ750458). The detection procedure was validated using a standard curve using a fragment of 16S rRNA from ‘*Ca*. L. asiaticus’ as template during the validation (S2 Fig). Five standards from affected tree and non-affected tree were also used as control. Each DNA sample tested in real time PCR was performed in triplicate and the quantification was analyzed with Rotor—Gene 3000 software (Corbett, Australia). All the qPCR products generated were sequenced to validate the identity to 16S rRNA from ‘*Ca*. L. asiaticus’ (S2 Fig). The resulting cycle threshold (Ct) values were converted to the estimated bacterial titers using the regression equation *y* = 10.501–0.309*x*, where ‘*y’* is the estimated log concentration of templates and ‘*x’* is the qPCR Ct values (R^2^ = 0.999).

### Transmission electron microscopy analysis

Transmission electron microscopy was carried out as a complementary analysis of HLB-affected 2-year-old ‘Valencia’ sweet orange plants treated and untreated with eBL at 0 and 12 months after treatment under greenhouse conditions. Fresh midrib samples (size: 5 x 2 mm) were fixed in 5% glutaraldehyde buffered at 4°C overnight. The samples were post-fixed with 1% osmium tetroxide for 12 hours at 4°C. The samples were then washed with 0.1 M sodium cacodylate buffer, pH 7.4. All samples were dehydrated sequentially with acetone (20, 30, 40, 50, 60, 70, 80 and 90%), for 15 min in each concentration at 4°C and finally for 1 hour in 100% acetone at room temperature. After dehydration the samples were infiltrated and embedded in Epon 812. After polymerization, ultra-thin sections were prepared with a diamond knife and ultra-microtome (NOVA, LKB) and placed on 400 mesh grids, stained with saturated uranyl acetate and lead citrate, and examined with a JEOL/JEM 2000 EX transmission electron microscope (JEOL, Japan). More than 100 microphotographs were searched for typical ‘Ca. L. asiaticus’ structures per treatment.

### Quantification of citrus defense gene expression

Total RNA was extracted from leaves of 2-year-old ‘Valencia’ sweet orange plants at 0, 1, 5 and 10 hours after treatment with eBL or water under greenhouse conditions. The concentrations of eBL were 0.084, 1.0, and 10 μM. The canopy was sprayed as described above. A total of 10 leaf samples were collected from five different positions around the tree canopy. The samples were immediately placed in liquid nitrogen. RNA was extracted using the RNeasy Plant Mini kit (Qiagen, Maryland, USA) and cDNA was synthesized using an oligo-(dT) primer and the SuperScript III reverse transcriptase kit (Invitrogen) according to the manufacturer’s instructions. Quantitative real-time PCR was conducted using a Rotor—Gene 3000 PCR machine with the QuantiTect SYBR Green PCR Kit. Primer sequences from known citrus defense genes were designed according to Primer 3 online software (S1 Table). Real-time PCR conditions were as follows: an initial 95°C denaturation step for 15 min followed by denaturation for 15 sec at 95°C, annealing for 30 sec at 60°C, and extension for 30 sec at 72°C for 40 cycles. PCR products were analyzed using the Rotor—Gene 3000 software. The housekeeping gene used for normalization was the citrus elongation factor 1-alpha; real-time quantification of target gene and housekeeping gene were performed in separate reactions. The threshold cycle (Ct) values were used to calculate the accumulation of target gene relative to elongation factor 1-alpha transcript by the 2 delta Ct method, where delta Ct = (Ct of target gene—Ct of elongation factor 1-alpha) [30]. Results were based on the average of at least three replicate reactions per sample. RT-PCR data were normally distributed as determined using the Ryan Joiner test within Minitab (Minitab release 13.32, Minitab Inc.). The significance of treatment effects was analyzed with Statistical Package for the Social Sciences (SPSS 11.0, SPSS, Inc., Chicago) as normally distributed data using one-way analysis of variance with post hoc pairwise least significance difference comparisons, *P <* 0.05.

### Construction of SuperSAGE libraries

Two SuperSAGE (serial analysis of gene expression) libraries were constructed to generate transcripts that were differentially induced in leaves of HLB-affected 2-year-old cultivar ‘Valencia’ sweet orange plants treated or untreated with eBL under greenhouse conditions. Leaves were collected from citrus plants treated with eBL (0.084 μM) at 1, 5 and 10 hr post-treatment and pooled before RNA extraction (treated library). As control, leaves from water-treated plants were harvested at the same time points (untreated library). Five replicates were used for each library. The samples were immediately placed in liquid nitrogen. Total RNA was extracted using the RNeasy kit (Qiagen, Maryland, USA), including an on-column DNAse treatment (Qiagen, Maryland, USA) according to the manufacturer’s instructions. The Super-SAGE library was constructed according to Matsumura *et al*. [31]. Double-stranded cDNA was synthesized using the biotinylated adapter-oligo dT primer (5’-bio-ctgatctagaggtaccggatcccagcag (t)^17^-3’). Purified cDNA was digested with anchoring enzymes NlaIII, the resulting fragments were bound to streptavidin-coated beads (Dynabeads streptavidin M-270), and non-biotinylated cDNA fragments were removed by washing. Adapter-2 (5’-caagcagaagacggcatacgatctaacgatgtacgcagcagcatg-3’ and 5’-ctgctgcgtacatcgttagatcgtatgccgtcttctgcttg-amino-3’) were ligated to cDNA fragments on the beads and after washing digested with EcoP15I. EcoP15I-digested and released fragments (adapter-2- tags) were ligated to adapters-1 (5’-acaggttcagagttctacagtccgacgatcxxxx-3’ and 5’ nnxxxxgatcgtcggactgtagaactctgaacctgt-amino-3’ (xxxx encodes variable index sequences) with defined index sequences for sample identification. Tags sandwiched between two adapters were amplified by PCR using Phusion High polymerase (New England Biolabs Inc. Ipswich, MA) and GEX primers (5’-aatgatacggcgaccaccgacaggttcagagttctacagtccga-3’ and 5’-caagcagaagacggcatacga-3’). The PCR reaction conditions were 98°C for 1 min, 3–10 cycles at 98°C for 30 sec, and 60°C for 30 sec. Eight tubes from this PCR amplification (each 15 μl) were pooled and the PCR products were concentrated using a MinElute reaction purification kit (Qiagen) and analyzed on an 8% non-denaturing polyacrylamide gel.

After staining with SYBR green (Takara Bio), the band at 123–125 bp was cut from the gel and the DNA purified from the gel pieces. PCR products from each sample were analyzed on an Agilent Bioanalyzer 2100 (Agilent Technologies, Santa Clara, CA). Equal concentrations of purified PCR products from the samples were mixed and sequenced with an Illumina Genome Analyzer II. Sequencing reactions used the GEX (DpnII) primer according to the manufacturer’s instructions. For each library, the tags were extracted from the sequences using the GXP- Tag sorter software provided by GenX Pro GmbH (Frankfurt am Main, Germany).

### Identification and functional annotation of differentially expressed SuperSAGE tags

Perl scripts were used to determine the unique sequences from both libraries and to remove singletons. A sequence was considered a singleton if it was detected only once in the combined libraries. Statistically significant changes in tag copy number between the untreated and treated sample libraries were analyzed by calculating a probability P-value. For fold-change (FC) calculations the libraries were normalized to 100,000 tags and the FC for each tag was calculated by dividing the number of tags in the treated sample library by the number of tags in the untreated library. Differentially expressed tags considered in this study were those having an FC equal or greater than 2.5 (up or down). Tag identification was performed by running Blastn against different databases [32]. *E*-value scores below 10^−5^ were considered as significant and were used to indicate homology between citrus sequences and database sequences. The task parameter was set to 'blastn-short’ in order to guarantee the optimal Blast functioning for short sequences searches. Blast2GO software was used to functionally annotate the UniTags. To confirm the SuperSAGE results, RT-PCR was carried out to compare the expression of identified UniTags. For this purpose, the 26-bp tag sequences obtained by SuperSAGE were directly used as 3’-RACE PCR primers to amplify the regions between the tag and poly-A tail. Using the same RNA, cDNA was synthesized separately using an anchored oligo dT primer (5’-biotin-ctgatctagaggtaccggatcccagcag (t)^17^–3’). For 3’- RACE PCR, 26-bp primers corresponding to the SuperSAGE tags were used in combination with the primer (polyT 5’-ggccacgcgtcgactagtac (t)^17^–3’) complementary to the cDNA ends. After amplification, products were separated in 1.5% preparative agarose gels. Bands corresponding to unequivocal amplicons were excised, and DNA was extracted with QIAquick cleanup columns (Qiagen, Maryland, USA). The PCR products were sequenced to confirm their identity.

## Results

### Reduction of ‘*Ca*. L. asiaticus’ bacterium titers under greenhouse conditions

**Fig 1:**
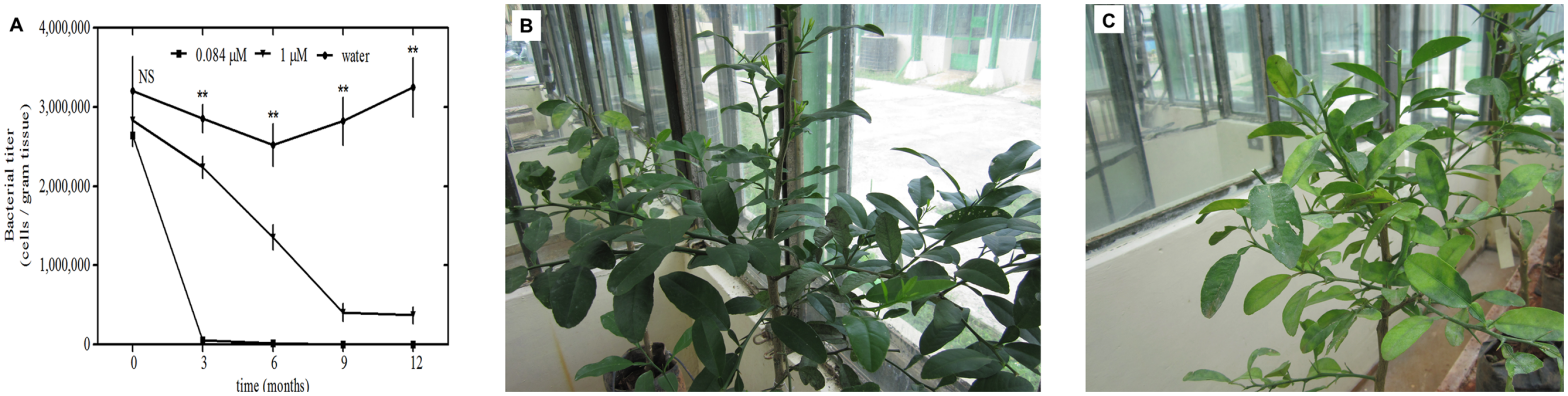
Induction of response of citrus plants to ‘*Ca*. L. asiaticus’ bacterium by eBL under greenhouse conditions. (A) Quantification of ‘*Ca*. L. asiaticus’ in HLB-affected citrus plants treated or untreated with eBL every 15 days for 12 months. Each point represents mean values with standard error (10 replicates per treatment). Significant difference among means was determined by two-way ANOVA least significant difference mean separation at *P *<* 0.05; **P <0.01; NS, not significant, F = 3.762. Data are representative of three independent experiments. Phenotype of HLB-affected citrus plants treated (B) and untreated (C) with eBL after 12 months.

eBL was tested for its activity against the ‘*Ca*. L. asiaticus’ bacterium in HLB-affected citrus plants in the greenhouse. Most plants treated with eBL showed a reduction in bacterial titers. Variance analysis indicated that the most effective concentration was 0.084 μM, with significant results seen at 3 months after treatments, compared with other concentrations and with untreated plants where the titers were stable throughout the time course. Foliar sprays every 15 days reduced bacterial titers to low levels, whereas after water treatment the bacterial titers were higher after 12 months (Fig 1A). Foliar application of eBL had a positive effect on the phenotype of the citrus plants, because new shoots appeared after 12 months. However, even with the treatment, the old leaves retained disease symptoms (Fig 1B). The estimated bacterial titers decreased from 2.6 x 10^6^ cells/g of plant tissue (prior to treatment) to 1.6 x 10^4^ cells/g of plant tissue (3 months after initial treatment with eBL), a 160-fold reduction (P<0.01; F = 3.762).

To study the anatomical changes caused by eBL treatment, we examined treated and untreated leaves from plants collected after 12 months using transmission electron microscopy. Untreated citrus plants contained a large number of bacterial cells of a pleomorphic shape in all samples of phloem sieve cells analyzed. Under higher magnification, the bacteria had the typical double membrane surrounding the cells. No bacteria were detected in plants treated with eBL (S3 Fig).

### Reduction of ‘*Ca*. L. asiaticus’ bacterium titers under field conditions

**Fig 2:**
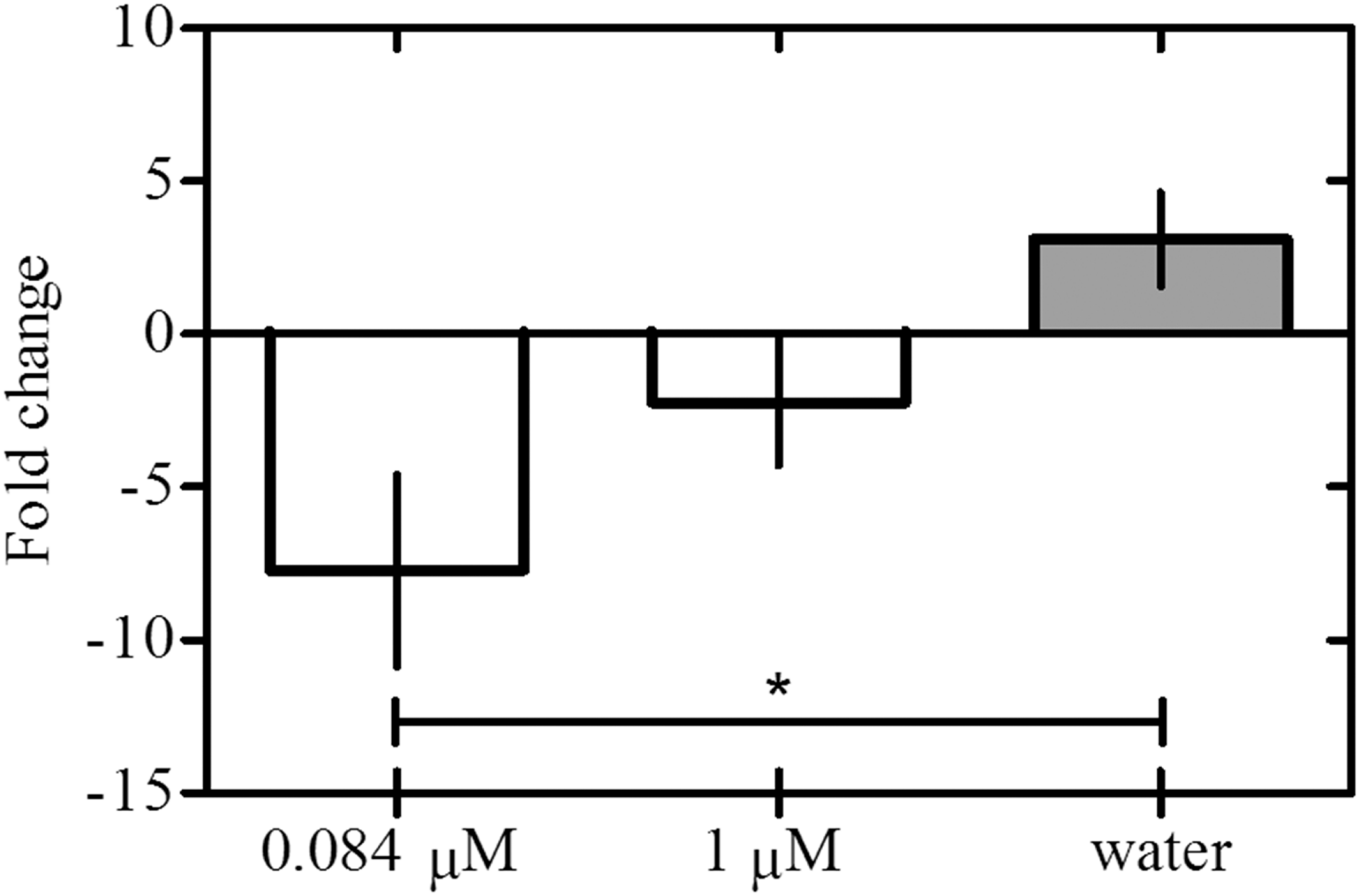
Induction of response of citrus plants to ‘*Ca*. L. asiaticus’ bacterium by eBL under field conditions. Fold change of ‘*Ca*. L. asiaticus’ titers in HLB-affected citrus plants treated or untreated with eBL after 12 months. The fold-change was calculated by dividing the average of total ‘*Ca*. L. asiaticus’ titers before the applications by the total ‘*Ca*. L. asiaticus’ titers after 12 months of applications. All the plants with and without typical symptoms of HLB prior to treatment were included in this analysis. Each point represents mean values with standard error (30 replicates per treatment). Significant difference among means was determined by one-way ANOVA least significant difference mean separation at *P *<* 0.05; F = 5.316.

To test whether application of eBL reduced the ‘*Ca*. L. asiaticus’ bacterial titers in the field, we tested two different concentrations of eBL (0.084 and 1.0 μM). When HLB-affected citrus trees were treated with 0.084 μM eBL in the field, the bacterial titers decreased more than 7-fold 12 months (P<0.05; F = 5.316) (Fig 2). In contrast, bacterial titers in untreated plants increased 3-fold over the same time period. Of the plants analyzed, 13 treated with 0.084 μM eBL had reduced titers after 12 months, whereas ten untreated plants showed increased titers (Table 1). At this concentration, two plants with symptoms of HLB prior to treatment had increased bacterial titers after 12 months, but the bacteria titer was only 4.7 x 10^4^ cells/g (Table 1).

**Table 1:** Summary of the status of HLB-affected citrus plants 12 months after treatment with eBL under field conditions.

Categories	eBL 0.084 μM	eBL 1.0 μM	Water
**Number of plants with typical symptoms of HLB prior to treatment**	15	15	15
**Number of plants with reduced bacterial titers after 12 months**	13	10	5
**Number of plants with increased bacterial titers after 12 months**	2	4	10
**Bacterial titer (cells / gram tissue) in plants with increased bacterial titers after 12 months**	4.7 x 10^4^	2.4 x 10^5^	3.4 x 10^6^
**Number of plants without typical symptoms of HLB and with no detectable bacteria prior to treatment**	15	15	15
**Number of plants without typical symptoms of HLB and no detected bacteria prior to treatment with increased bacterial titers after 12 months**	0	4	12

### Induction of defense-related genes by eBL

**Fig 3:**
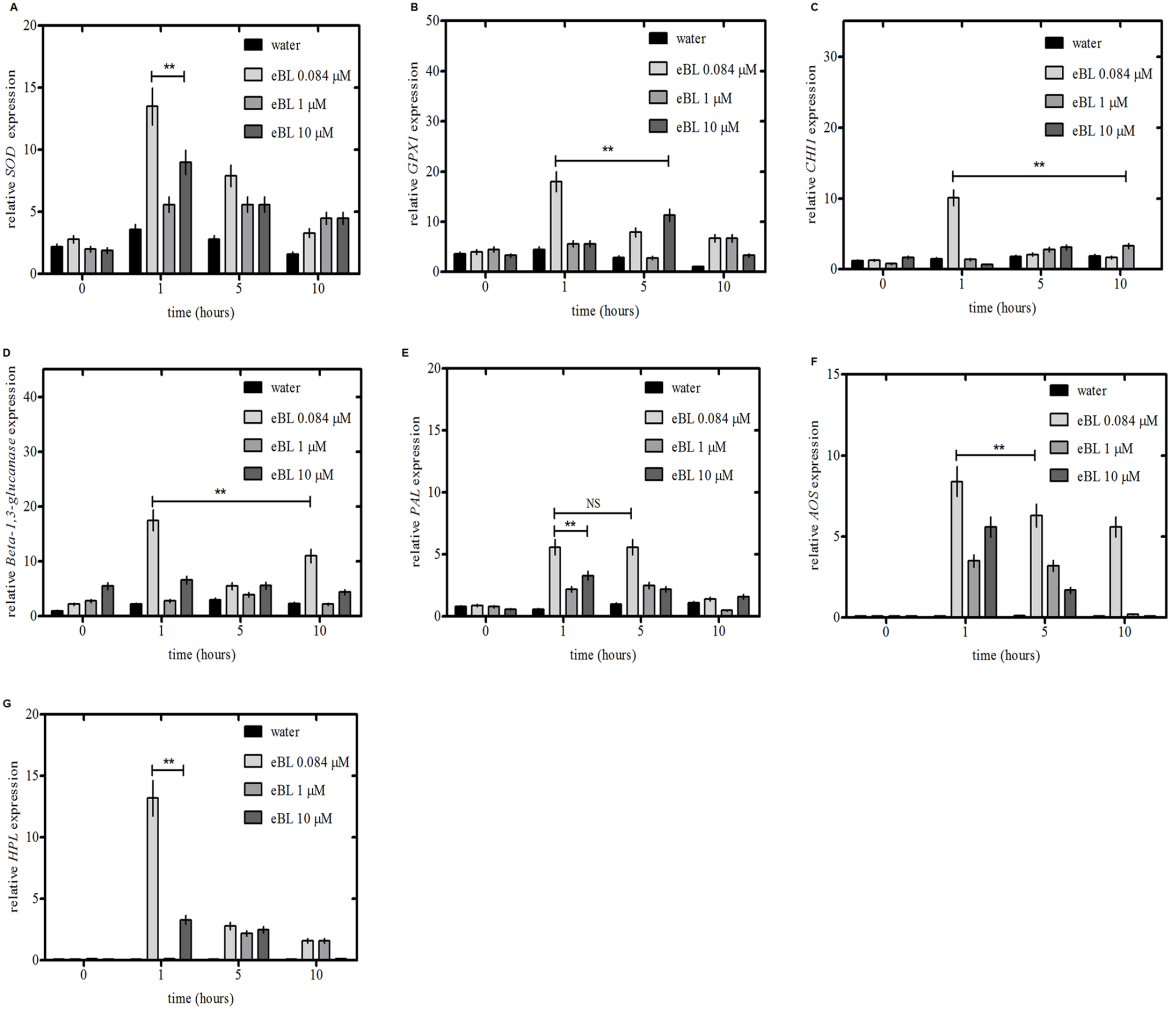
Induction of defense-related genes in HLB-affected citrus plants treated with eBL. (A) Superoxide dismutase (SOD), (B) Glutathione peroxidase (GPX1), (C) Chitinase (CHI1), (D) Beta-1, 3 glucanase, (E) Phenylalanine ammonia-lyase (PAL), (F) Allene oxide synthase (AOS), (G) Fatty acid hydroperoxide lyase (HPL). Accession numbers are given in S1 Table. Results are based on three experiments, each with three replicates per treatment. Bars indicate standard error of the mean and least significant difference at *P *<* 0.05; **P <0.01; NS, not significant.

We evaluated the relative expression of defense-related genes using qPCR. Genes for superoxide dismutase (SOD), glutathione peroxidase (GPX1), chitinase (CHI1), beta-1, 3 glucanase, phenylalanine ammonia-lyase (PAL), allene oxide synthase (AOS) and fatty acid hydroperoxide lyase (HLP) were significantly induced after one hour compared to untreated plants (P<0.01) (Fig 3). Interestingly, expression of genes for *SOD*, *GPX1*, *CHI1*, beta-1, 3 glucanase, *AOS* and *HLP* were reduced after one hour. Expression of the gene for *PAL* decreased significantly after five hours. The induction of the defense-related genes was linked to the concentration of eBL tested. A concentration of 0.084 μM eBL induced the highest transcripts levels (Fig 3).

### Induction of new defense related genes determined by SuperSAGE

To look for new genes induced by eBL treatment in HLB-affected citrus plants, a transcript profile analysis was conducted using SuperSAGE libraries combined with sequencing. Leaves treated or not treated with eBL were collected at 1, 5, and 10 hr following foliar application. The total number of SuperSAGE tags obtained after sequencing the libraries and eliminating incomplete reads, twin-ditags, and ditags without complete library-identification DNA linkers was 781,750, of which 601,748 tags were from the treated library and 180,002 from the untreated library. These tags represented 113,283 unique sequences (47,747 in the untreated library and 65,536 in the treated library) (Table 2). These tags are referred to as UniTags and were further analyzed. Statistically significant changes in tag copy number between the untreated and treated sample libraries were analyzed by calculating a probability (P)-value. Although small changes in expression levels may have biological significance, we focused primarily on genes which showed changes in expression levels greater than 2.5-fold. Based on the calculated (P)-values and using a 95% confidence level, 13,036 UniTags were identified as differentially expressed in plants treated or untreated with eBL. Among these UniTags, 10,505 were up-regulated by eBL.

**Table 2:** Summary of the SuperSAGE libraries.

Library	untreated	treated	total
**Sequenced tags**	180,002	601,748	781,750
**Number of unique transcripts (UniTags)**	47,747	65,536	113,283
**Differential expressed “Tags”**	2531	10505	13036
**Function according to Gene Ontology**	259	643	902

To obtain gene function categories of the differentially expressed UniTags, gene ontology (GO) annotation of the nucleotide sequences was performed by BLASTX against the non-redundant GenBank and UniProtKB/TrEMBL protein databases. For this analysis, we used UniTags that showed a maximum of two mismatches (24/26) with entries in the GenBank nucleotide database. Of the differentially expressed UniTags, a total of 643 from the treated library and 259 from the untreated library had FC values ≥ 2.5 (Table 2). The largest set of these genes was assigned to oxidation/reduction, while genes involved in cellular response to deprivation constituted the smallest group. Genes involved in defense response to bacteria formed the eighth biggest group (S4 Fig). Of the 643 and 259 “UniTags” from treated and untreated libraries, respectively, 63 were common between these libraries. Most relevant to our study, 24 and 26 “UniTags” were genes classified as defense or hormone-related in the treated and untreated libraries, respectively. Meanwhile, six “UniTags” were common between treated and untreated libraries with respect to defense response and hormone pathway genes (S5 Fig).

Based on previous knowledge regarding the influence of eBL on defense responses and the pathways of non-brassinosteroid hormones, we focused further analyses on the genes included in these categories. By this analysis, several new genes involved in defense response to the bacterium, response to jasmonic acid, and response to salicylic acid were identified in affected citrus plants treated with eBL, which suggests that that they are involved in eBL-regulated defense response (Table 3). Some of these genes were up-regulated by eBL treatment and some were down-regulated; however the expression levels were on average higher in the group of up-regulated genes (S6 Fig).

**Table 3:** Differentially expressed genes in citrus plants treated with eBL.

Tag-Id	Tag sequence	FC ^a^	Protein Identification
**Tag_9379**	CATGGAAGAGTTGCAAGCTGCAATTC	3.92	bri1-kd interacting protein 119
**Tag_44273**	CATGACTGCATATGTAATCTGCATAA	4.8	glucosyl transferase
**Tag_438**	CATGCACAAGCAAATGCTGGCGCCCC	-2.6	bax inhibitor protein 4
**Tag_10875**	CATGGATTGGTTGAATTTCAAAATTA	-6.9	brassinosteroid insensitive 1-associated receptor kinase 1
**Tag_37209**	CATGACTGCATATGTAATCTGCATAA	3.7	arp protein
**Tag_1711**	CATGTGAGCAGGTTTCAAACTGCATA	28.3	lethal leaf spot 1-like protein
**Tag_18558**	CATGCAATCTCTTGTATTGCAAGTAG	34.6	myo-inositol 1-phosphate synthase
**Tag_43758**	CATGGCTGAGCTGGCGCTCATTGGGT	6.0	natural resistance-associated macrophage protein
**Tag_21737**	CATGCTGACTTGGAACAGCATAGGGT	15.4	nramp transporter
**Tag_5170**	CATGGCGCGCCTATGTGCAATTTTAC	3.5	neutral alpha-glucosidase neutral alpha-glucosidase
**Tag_29001**	CATGCATCCCGAGGAATTGTAAATAC	4.0	tga1 transcription factor
**Tag_19370**	CATGTGCAACAAGTAGCAGGATGGGG	8.6	tryptophan synthase alpha chain
**Tag_20583**	CATGGGCACTACAGTTATGTAATAAA	46.1	wound-induced protein win1
**Tag_32487**	CATGGATCAATGAGTCTTCTCCAAAA	8.3	acidic class II chitinase
**Tag_5495**	CATGCCACGATGTTATCACAGGCCAA	8.3	chitinase class I
**Tag_46933**	CATGTTACTTATTGTGGATACATAAG	4.0	cold-regulated ltcor12
**Tag_49947**	CATGGTTGATCTCAGGATCTGTGATG	4.0	defensin 1
**Tag_46675**	CATGGGCTTCTGACTCCAGACTGGCC	4.8	gibberellin-regulated protein 1
**Tag_1369**	CATGAATGAATAAAATGTGTTACTCT	9.1	NBS-LRR resistance protein
**Tag_31398**	CATGATGATGCGGTTCAGCCTGTAAA	3.1	protein kinase chloroplast
**Tag_24302**	CATGTAGTTATGTGCGTGAATTAAAA	23.5	thaumatin-like protein
**Tag_48298**	CATGAATCGTGTTCCTTAACAAAAAC	23.5	protease inhibitor
**Tag_11640**	CATGGCCTACCGGCTGCTGATTGGAA	-1.8	DNA-binding protein
**Tag_7057**	CATGGAAGTGGATTGACTTTTGGCTT	-10.4	heat shock protein 83
**Tag_2649**	CATGTTTGGAGTAGGAATGTTGTTCG	-27.9	histone 2
**Tag_4540**	CATGCTCAAAGCTCTGTGATTTCGGT	-2.91	peroxiredoxin-like protein
**Tag_13567**	CATGCCTTAACAGCTGTTGGATCCGG	-1.8	proteasome subunit alpha type 2
**Tag_3863**	CATGTCCTCTGCTATCACCGGAGACG	-5.4	putative DNA-binding protein
**Tag_5445**	CATGATGAATCAGTTTGGATTTTGTA	-13.9	rar1 protein
**Tag_730**	CATGGCCCTCGGCCTCGGTACCGGCT	-22.7	ribose-5-phosphate isomerase
**Tag_7866**	CATGAAGGTAGATATCACAGGAAATC	-2.7	ribosome recycling factor
**Tag_3234**	CATGGAAGCAACCAAATTGGAAGTAA	-2.7	spl1-related2 protein
**Tag_4283**	CATGAGTTTTTAGTTCTGGCTTCTGA	-4.5	proteasome subunit alpha type 7
**Tag_8415**	CATGTAGTTACAGGGGCTCAGCTTGG	-5.2	mac perforin domain containing protein
**Tag_11336**	CATGAAGATCAATGGAGATTGATGTT	-1.8	jaz10
**Tag_1587**	CATGTTCGAGGCGCCATCTGTGGCTG	-1.8	nad-dependent epimerase dehydratase
**Tag_9103**	CATGGTGCGGCTGTTGTGGGCTGACG	-2.7	med21
**Tag_4578**	CATGCTCCAACTGGAGACTTGGTGGA	-3.4	pathogenesis-related protein
**Tag_1659**	CATGGACGGCGTCGCGTATTCCAGCA	-10.9	peptidase M
**Tag_19328**	CATGTAACAACTTGCAAACGTGTCAA	5.1	NADPH-ferrihemoprotein reductase atr1
**Tag_22324**	CATGCACAAGCAAATGCTGGCGCCCC	76.5	phosphoribosylanthranilate transferase
**Tag_29653**	CATGCATTTTTTAACTGGGTTCCATT	3.7	polyubiquitin-like protein
**Tag_16442**	CATGAAAACCTGATTGGTTGTTCCAA	4.5	histone deacetylase
**Tag_33**	CATGGCTTGGCCTGGCTGCTCAAACT	-2.5	jasmonate zim-domain protein 1
**Tag_13**	CATGGCTTGGCCTGGCCGCTCAAACT	-7.0	plastid jasmonate zim-domain protein
**Tag_13989**	CATGTTGCATTCACAGTGCTCTTCGT	-6.9	pdr12 (pleiotropic drug resistance 12)
**Tag_7622**	CATGCAAAGGACTGGTTAGTGAGTAA	-10.4	r2r3-myb transcription factor
**Tag_14534**	CATGCTGACGGGTATGCGTCGGAAGA	-1.8	myb family transcription factor
**Tag_3755**	CATGGATTGGTTGAATTTCAAAATTA	-6.9	3-ketoacyl- thiolase
**Tag_22336**	CATGATTATTAATTTGAAGCTTGGGC	11.7	acetone-cyanohydrin lyase
**Tag_12060**	CATGCCAGACACCAAACACCAACCAT	7.7	polyneuridine-aldehyde esterase

^a^ The libraries were normalized to 100,000 tags and the fold-change (FC) for each tag was calculated by dividing the number of tags in the treated sample by the number of tags in the untreated sample. Five replicate were used for each library.

The genes encoding a bri1-kd interacting protein, a glucosyl transferase, and glycogen synthase kinase-3 involved in the brassinosteroid signaling pathway increased in eBL-treated plants. The expression of a gene encoding the transmembrane bax inhibitor motif-containing protein 4 and brassinosteroid insensitive 1-associated receptor kinase 1 expressed were repressed by eBL treatment. Several genes encoding enzymes in jasmonic acid metabolism were up-regulated by eBL, including NADPH—ferrihemoprotein reductase atr1, phosphoribosylanthranilate transferase, polyubiquitin-like protein, and histone deacetylase. Further, two genes, encoding an acetone-cyanohydrin lyase and polyneuridine-aldehyde esterase, which are related to response to salicylic acid biosynthesis, were induced. Interestingly, several other genes, not traditionally classified as involved in defense, were induced in plants treated with eBL. These included transcripts encoding actin related protein (ARP), glyceraldehyde-3-phosphate dehydrogenase, lethal leaf spot 1-like protein, myo-inositol 1-phosphate synthase, natural resistance-associated macrophage protein, neutral alpha-glucosidase, nramp transporter, tga1 DNA binding calmodulin binding transcription factor, tryptophan synthase alpha chain, wound-induced protein win1, defensin 1, gibberellin-regulated protein 1, NBS-LRR resistance protein, protein kinase chloroplast, thaumatin-like protein and protease inhibitor, respectively (Table 3).

## Discussion

Most citrus-producing countries are severely affected by HLB, the most devastating disease of citrus plants [3]. The disease has not been eradicated from any region where infection has been reported. Although the curative effects of different antibiotic combinations have been evaluated in the greenhouse, further evaluation of the cost and environmental effects is essential to establish the practicality of this approach [33]. Additionally, the use of enhanced nutritional programs to minimize the deleterious effects of HLB has been evaluated, but this approach did not consistently enhance tree health, yield, or fruit quality [9]. Thus, there is currently no established effective control of citrus HLB. We report here the first study by which the bacterial titers in affected citrus plants decreased significantly following foliar spraying with eBL, in both the greenhouse and the field. Additionally, we showed that eBL induces defense genes in citrus plants affected with HLB. In field trials, we observed a reduction of ‘*Ca*. L. asiaticus’ titers in HLB-affected citrus plants after treatment with eBL every 15 days for 12 months. eBL might therefore be a useful tool for the management of ‘*Ca*. L. asiaticus’ in integrated programs. There was a very dramatic reduction of bacteria titers in greenhouse conditions compared with field conditions. The control of environmental parameters such as temperature, humidity, rain and other factors and lack of vector to re-infect in greenhouse conditions might have contributed to the more dramatic effects seen in the greenhouse (S7 Fig).

Brassinosteroids induce disease resistance in tobacco, rice, cucumber, potato, tomato and barley plants against bacteria, fungi, oomycetes and viruses [20–28]. For example, treatment of potato plants under field conditions with 24-epibrassinolide decreased the level of infection by *Phytophthora infestans*, and in some cases the protection was higher than for plants treated with standard fungicides [20]. However, the induction of resistance by eBL in citrus already infected with HLB disease has not previously been documented.

eBL induced a number of genes in citrus, which might contribute to the protective effects of eBL. For example, the level of transcripts of GPX1 encoding a glutathione peroxidase transcript peaked at 1 h after treatment with 0.084 μM eBL. Glutathione peroxidases are a family of enzymes involved in hydrogen peroxide (H_2_O_2_) and lipid reduction to protect cells against oxidative damage. This is probably related to the production of H_2_O_2_ which is one of the first plant responses to stress [34]. Expression levels of some genes associated with systemic acquired resistance (SAR), such as chitinase and beta1, 3-glucanase, were enhanced by eBL treatment [34]. Another induced gene encoded allene oxidase synthase, a key enzyme in the jasmonate acid (JA) synthesis pathway and known to be involved in defense gene activation [35, 36].

Jasmonate acid plays an essential role in induced systemic resistance (ISR) [35, 36]. ISR depends on the timely accumulation of multiple gene products including fatty acid hydroperoxide lyase (HPL), allene oxide synthase (AOS), and phenylalanine ammonia lyase (PAL). After 1 h of eBL treatment, all three (HPL, AOS, and PAL) showed significantly higher expression, and the expression levels of all three were higher in treated plants at all-time points. The role of PAL in broad-spectrum pathogen defense is well-known, and transcription of the gene encoding PAL is activated by the JA/ethylene signaling pathway during induced systemic resistance [37–39]. Enhanced expression of these three genes, known to be involved in ISR, might be one of the reasons for the increased resistance of eBL-treated plants to ‘*Ca*. L. asiaticus’.

Our findings suggest that the ability of the host to effectively up-regulate defense gene expression results in control of bacterial infection. Interestingly, a large number of genes involved in plant defense and the salicylic acid and jasmonic acid pathways were induced in HLB-affected citrus trees, demonstrating that even susceptible hosts initiate defense responses, although apparently insufficiently to restrict multiplication and spread of the pathogen throughout the plant [40–45].

Genes related to the salicylic acid response were also induced by eBL treatment. Interestingly, ‘*Ca*. L. asiaticus’ encodes a functional salicylate hydroxylase (SahA) that converts salicylic acid into catechol [46]. Plants that are unable to accumulate salicylic acid through the transgenic expression of a bacterial salicylate hydroxylase (NahG) that metabolizes salicylic acid into catechol are deficient in expression of PR genes and in SAR [47, 48]. Therefore, SA responses are probably not important for resistance to ‘*Ca*. L. asiaticus’, and therefore eBL treatment is working through different pathways.

Of particular interest, several genes encoding components of the brassinosteroid signaling pathway were up-regulated in citrus plants treated with eBL. However, their connections to HLB regulation of SA-ET-JA crosstalk remain to be determined. The brassinosteroid and PAMPs signaling modulate each other through BAK1. Brassinosteroid and flg22 each induce distinct biochemical and gene expression responses, without detectable overlap. When plants are treated with BR and flg22, flg22 has no effect on brassinosteroid induced responses, but brassinosteroid significantly decreases flg22-induced PTI responses, including oxidative burst and defense gene expression [49]. These results suggest a unidirectional brassinosteroid inhibition of PTI responses, whereas flg22 has no effect on brassinosteroid-induced responses [49]. In contrast to this, the report by Belkhadir *et al*. [17] provides evidence for brassinosteroid modulation of PTI responses through both dependent and independent mechanisms.

## Conclusion

In this study, we report that the application of epibrassinolide reduces the causal agent of HLB and might provide a useful tool within an integrated management program. Mostly, plants with ‘*Ca*. L. asiaticus’ treated with eBL showed a marked reduction in bacterial titers. When the ‘*Ca*. L. asiaticus’-affected plants were treated with eBL at concentration of 0.084 μM, titers were drastically reduced after three months, compared with untreated plants where the titers were stable throughout the time course under greenhouse conditions. The results suggest that eBL treatment dynamically increased the transcripts of an array of defense genes in the citrus plant against the ‘*Ca*. L. asiaticus’ bacterium. Several defense response genes are highly activated after eBL treatment. These findings suggest an ability of the host to effectively enhance defense resulting in the reduction of the bacterial titers in the plant. The application of eBL holds promise to improve the innate immune response to this pathogen and change the environment to reduce the multiplication and spread of ‘*Ca*. L. asiaticus’. Finally, the influence of eBL application on several parameters such as disease symptomatology, citrus physiology, and yields should be studied to better understand the complexity of citrus HLB.

## Supporting Information

S1 Fig Experimental design used for the evaluation of eBL under field conditions. Shaded column represents citrus trees treated with water (A), 1 μM (B) or 0.084 μM eBL (C) every 15 days for 12 months (N = 148 treated plants per treatment). The final solution was 232 liters per hectare, approximately 1.34 liters per plant in each application. Black boxes represent the plants with typical symptoms of HLB and with detectable bacteria prior to treatment randomly selected to test the dynamics of bacterial titers every three months (N = 15 plants per treatment). Empty boxes symbolize the plants without typical symptoms of HLB and with no detectable bacteria prior to treatment randomly selected to test the dynamic of bacterial titers every three months (N = 15 plants per treatment). The bacterial titers were determined for all plants per treatment prior to the applications of eBL.
(TIF)

S2 Fig Validation report for quantification of ‘*Ca*. L. asiaticus’ using real time -PCR. (A) Information, data, raw data and standard curve of quantification using SYBR Green. The standard curve was used in each bacterial titers determination. (B) A fragment of 16S rRNA from ‘*Ca*. L. asiaticus’ used as template during the validation and titers determination is shown in green. The primer sequences are shown in red font underline. All the PCR products generated during bacterial titers determination were sequenced and validated according the sequence of 16S rRNA. (C) Validation of positive (plants with typical symptoms of HLB) and negative control (Mexican lime plants without typical symptoms of HLB obtained from seeds) used in each bacterial titers determination.
(TIF)

S3 Fig Transmission electron micrograph of ultra-thin sections of HLB-affected Mexican lime (*Citrus aurantifolia*) leaves treated (A) or untreated (B, C) with eBL after 12 months. HLB-affected citrus untreated showing the elongated and spherical forms bodies from ‘*Ca*. L. asiaticus’ (arrow).
(TIF)

S4 Fig Distribution of annotated UniTags in gene ontology categories. (TIF)

S5 Fig Venn diagram showing commonality of differentially expressed genes with respect to treated library, untreated library, defense response and hormone pathways genes and their interaction in citrus plants. (TIF)

S6 Fig Number of genes in treated and untreated samples with eBL belonging to the different categories. These genes were included in Table 2.
(TIF)

S7 Fig Climatic variables during the experiment under field conditions. The asterisk symbolizes the date of evaluation of the titers.
(TIF)

S1 Table List of primers used in real time (RT)-PCR reactions. (TIF)
