# Supplementary figures and images for: ‘Candidatus Liberibacter asiaticus’, Causal Agent of Citrus Huanglongbing, Is Reduced by Treatment with Brassinosteroids
Source: PLoS One. 2016 Jan 5;11(1):e0146223. doi: 10.1371/journal.pone.0146223 (PMC4701442; doi:10.1371/journal.pone.0146223)

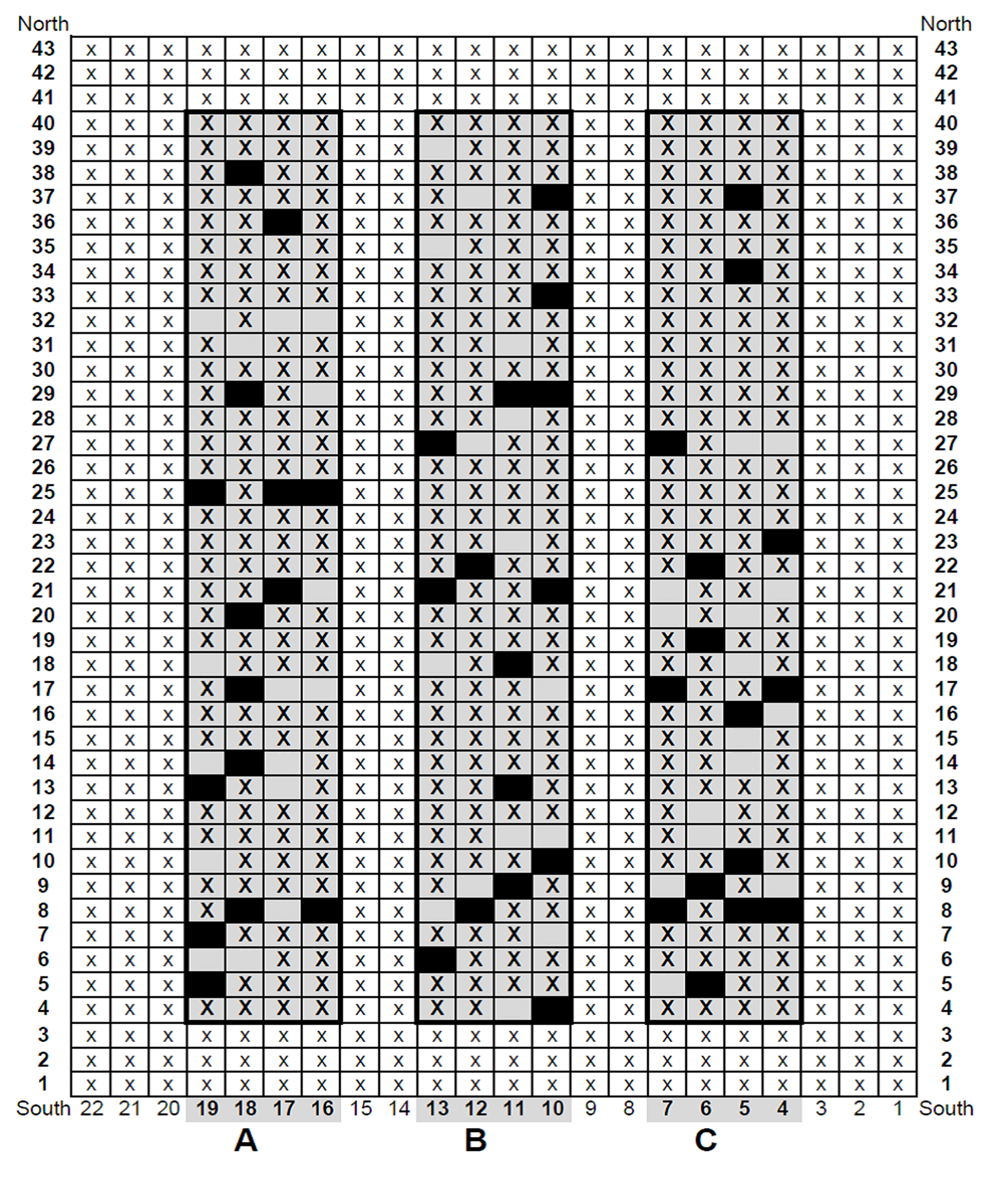

Supplement: S1 Fig — Shaded column represents citrus trees treated with water (A), 1 μM (B) or 0.084 μM eBL (C) every 15 days for 12 months (N = 148 treated plants per treatment). The final solution was 232 liters per hectare, approximately 1.34 liters per plant in each application. Black boxes represent the plants with typical symptoms of HLB and with detectable bacteria prior to treatment randomly selected to test the dynamics of bacterial titers every three months (N = 15 plants per treatment). Empty boxes symbolize the plants without typical symptoms of HLB and with no detectable bacteria prior to treatment randomly selected to test the dynamic of bacterial titers every three months (N = 15 plants per treatment). The bacterial titers were determined for all plants per treatment prior to the applications of eBL. (TIF) [file pone.0146223.s001.tif]

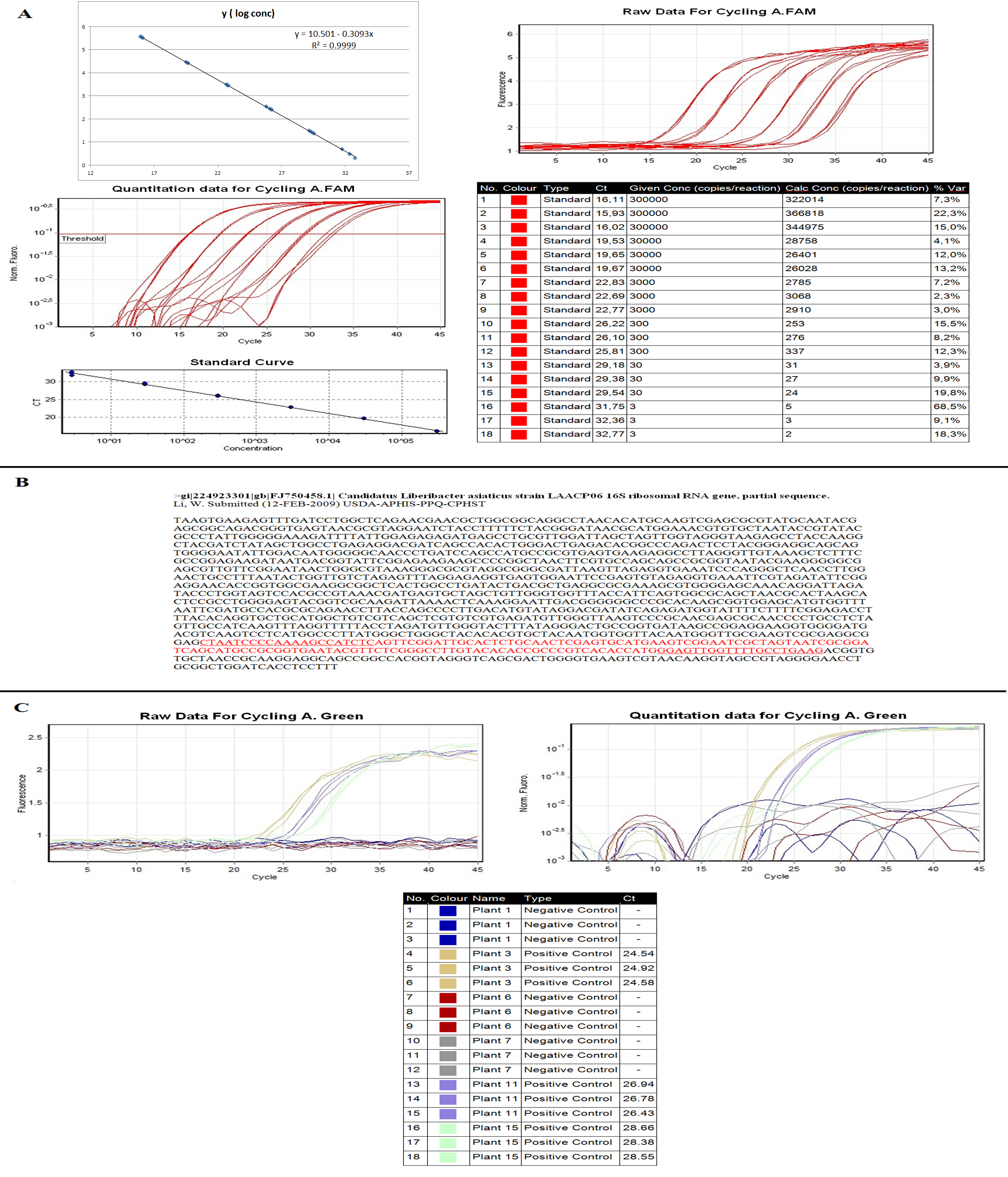

Supplement: S2 Fig — (A) Information, data, raw data and standard curve of quantification using SYBR Green. The standard curve was used in each bacterial titers determination. (B) A fragment of 16S rRNA from ‘Ca. L. asiaticus’ used as template during the validation and titers determination is shown in green. The primer sequences are shown in red font underline. All the PCR products generated during bacterial titers determination were sequenced and validated according the sequence of 16S rRNA. (C) Validation of positive (plants with typical symptoms of HLB) and negative control (Mexican lime plants without typical symptoms of HLB obtained from seeds) used in each bacterial titers determination. (TIF) [file pone.0146223.s002.tif]

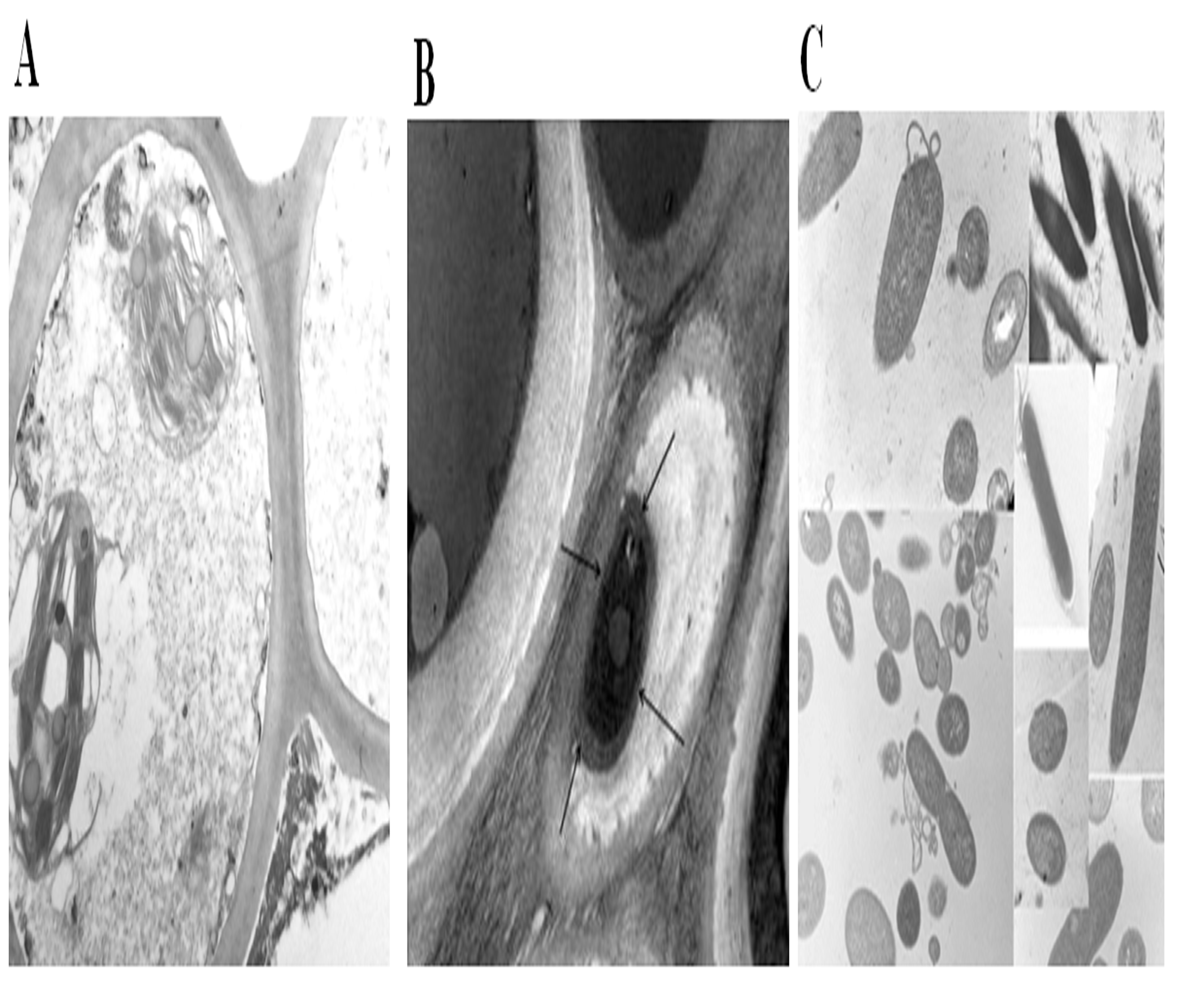

Supplement: S3 Fig — HLB-affected citrus untreated showing the elongated and spherical forms bodies from ‘Ca. L. asiaticus’ (arrow). (TIF) [file pone.0146223.s003.tif]

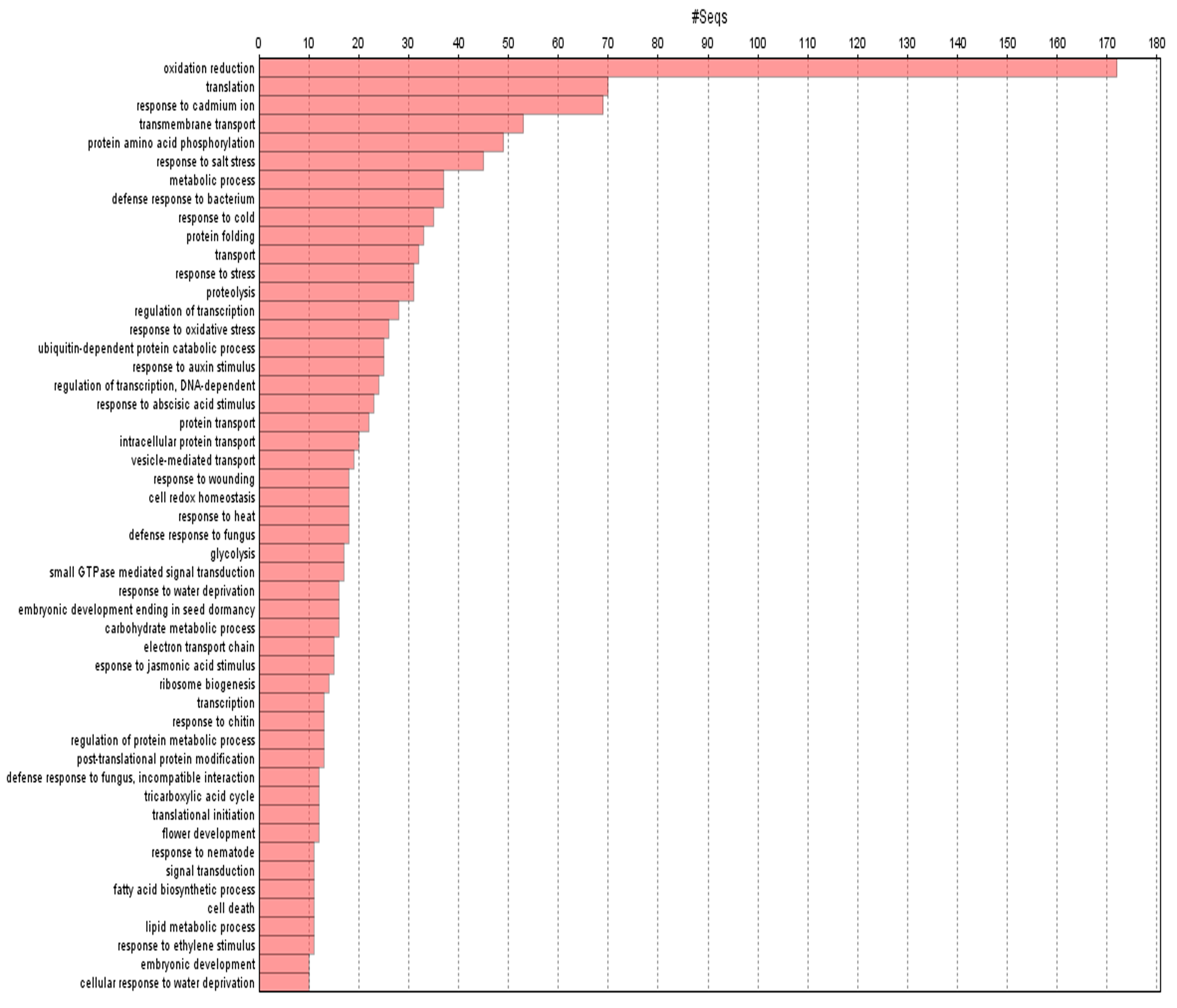

Supplement: S4 Fig — (TIF) [file pone.0146223.s004.tif]

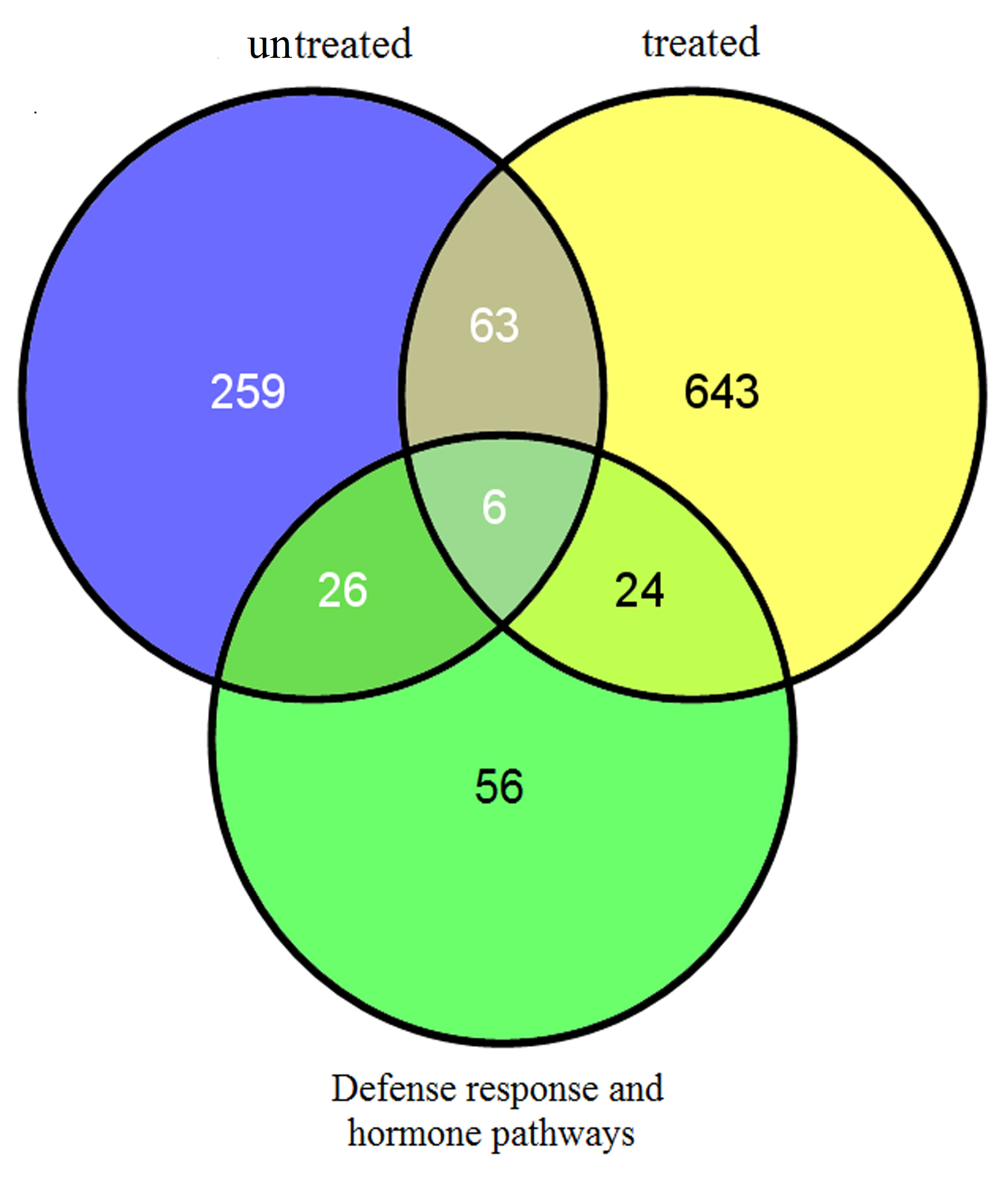

Supplement: S5 Fig — (TIF) [file pone.0146223.s005.tif]

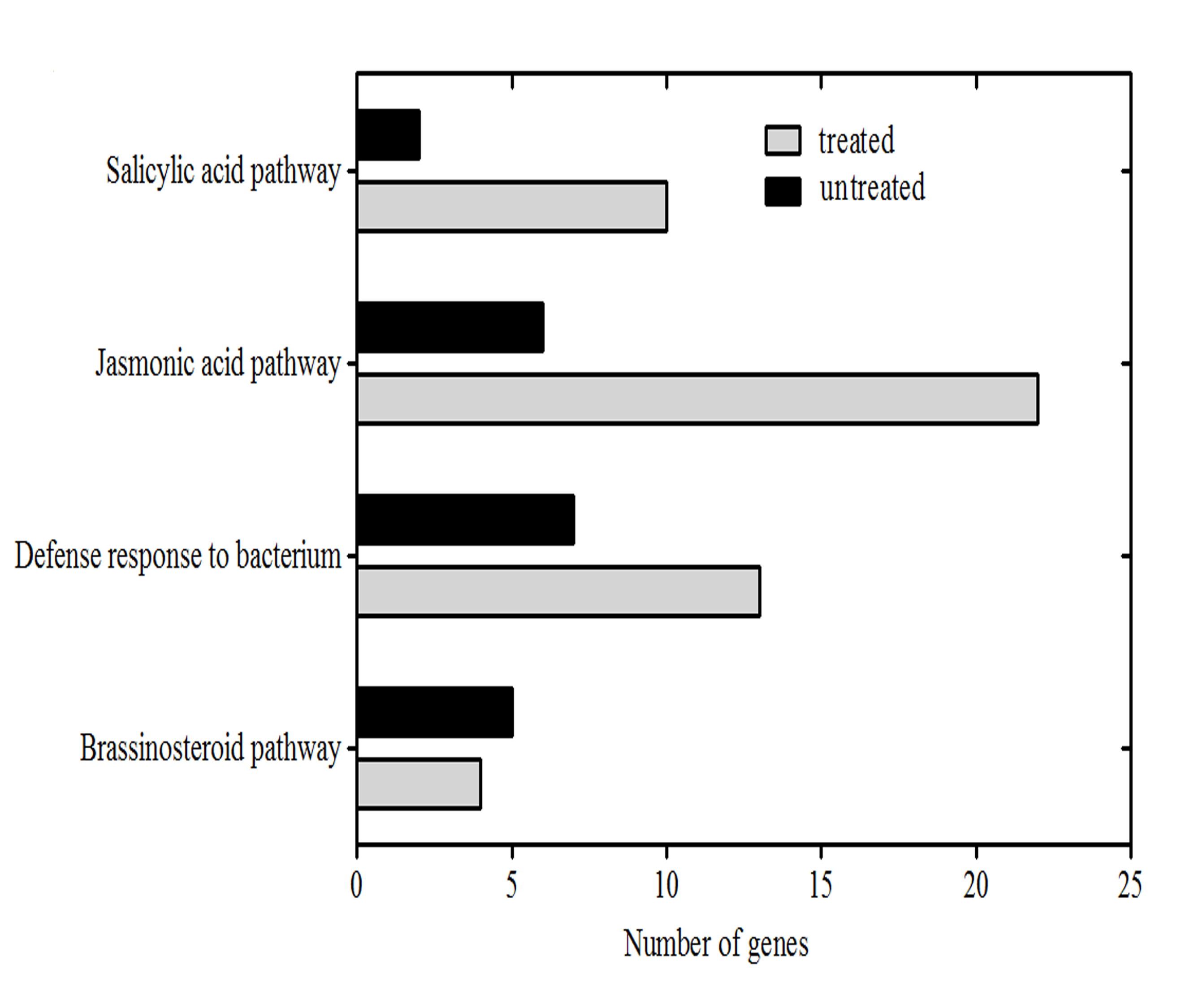

Supplement: S6 Fig — These genes were included in Table 2. (TIF) [file pone.0146223.s006.tif]

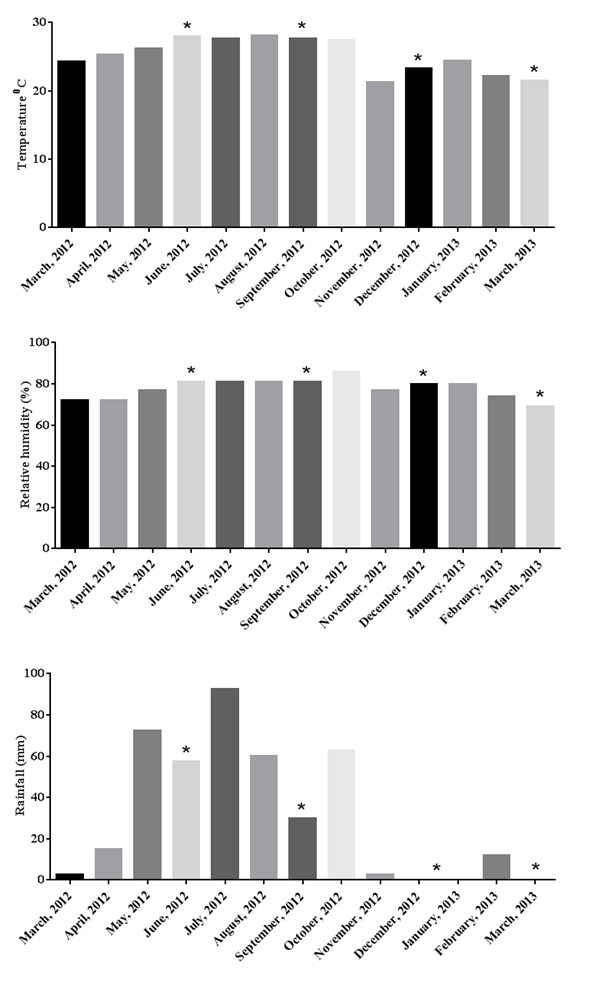

Supplement: S7 Fig — The asterisk symbolizes the date of evaluation of the titers. (TIF) [file pone.0146223.s007.tif]

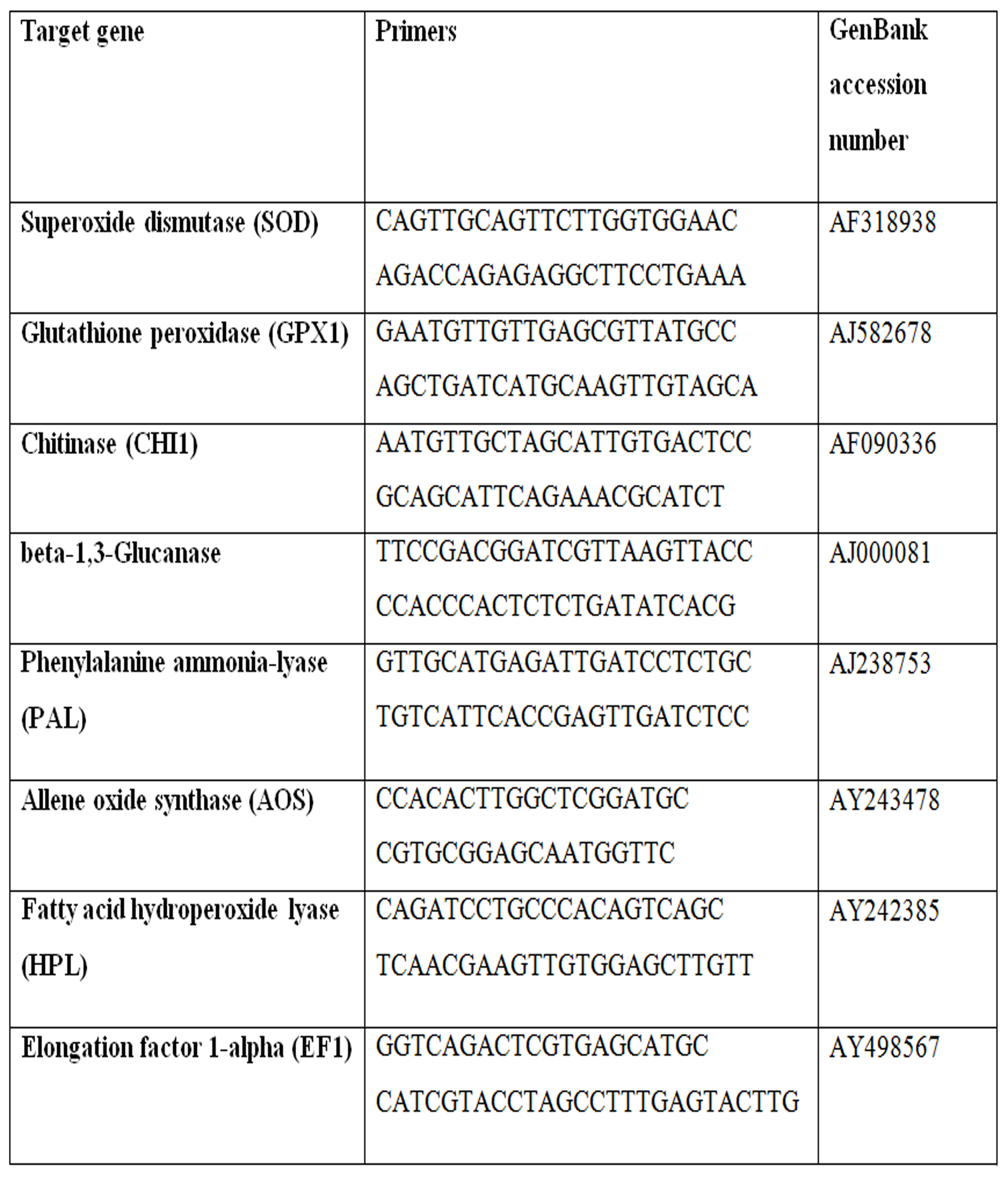

Supplement: S1 Table — (TIF) [file pone.0146223.s008.tif]
